# Supplementary material for: Structural Insights into the Chaperone-Assisted Assembly of a Simplified Tail Fiber of the Myocyanophage Pam3
Source: Viruses. 2022 Oct 14;14(10):2260. doi: 10.3390/v14102260 (PMC9608196; doi:10.3390/v14102260)
Supplement: Supplementary file 1 [file viruses-14-02260-s001.zip › viruses-1940026-supplementary.pdf]

Supplementary figures and figure legends

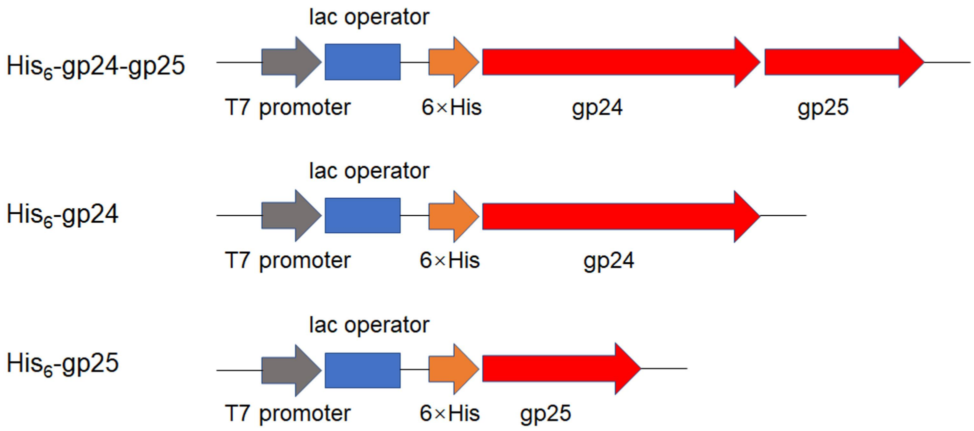

Figure S1. The vectors' architecture of *His$_6$-gp24-gp25, His$_6$-gp24 and His$_6$-gp25*.

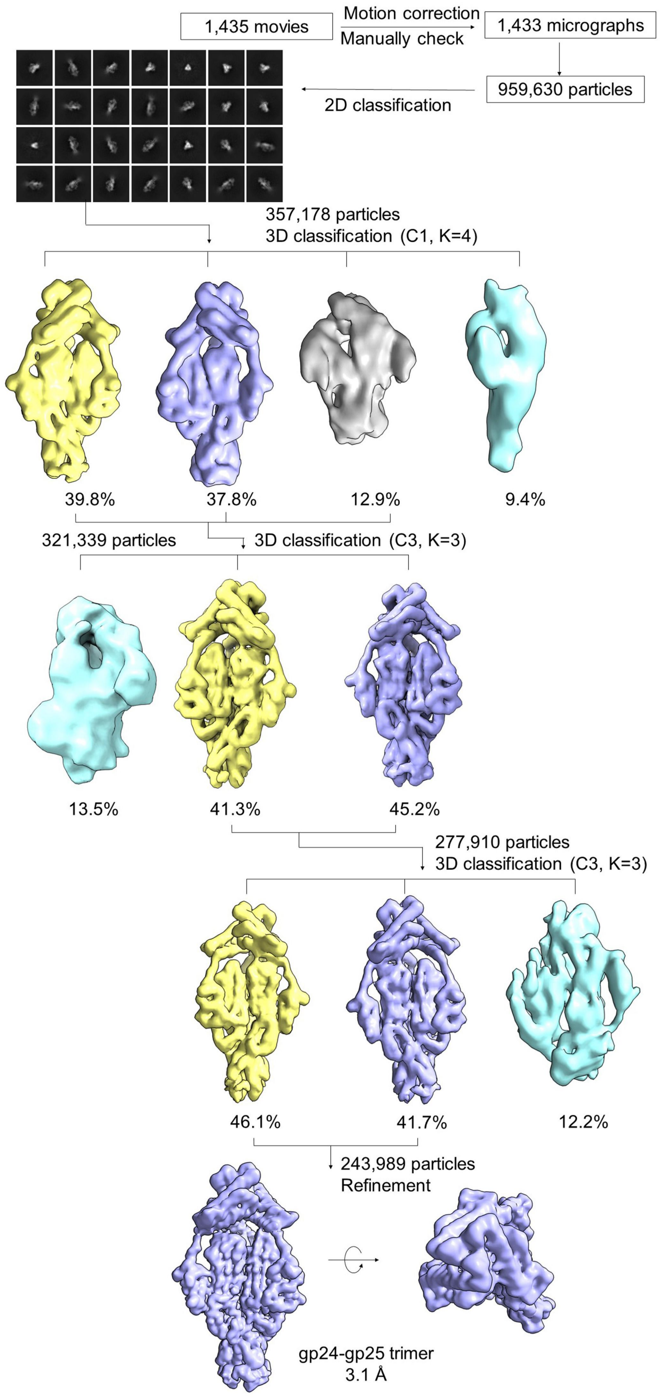

Figure S2. A flowchart of cryo-EM data processing of the gp24-gp25 complex.

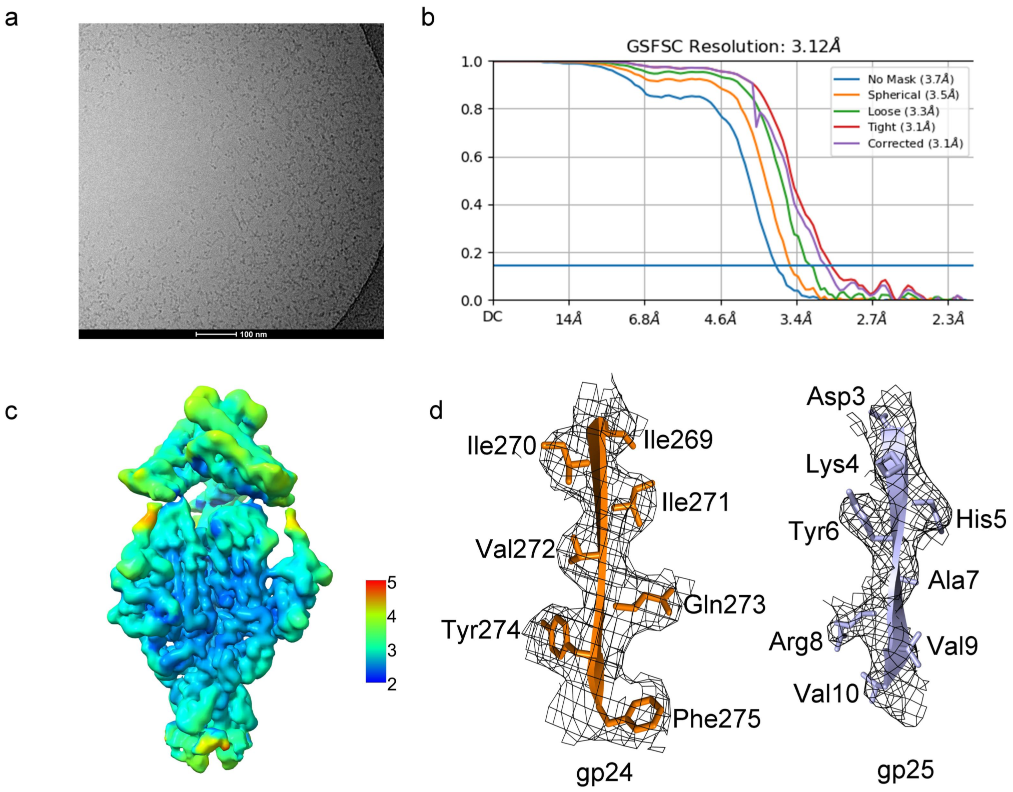

Figure S3. The cryo-EM data analysis of gp24-gp25 complex. (a) A representative raw micrograph of the gp24-gp25 complex. (b) The resolution curve of the cryo-EM maps estimated using the gold-standard Fourier shell correlation at 0.143 criterion. (c) The local resolution map of the gp24-gp25 complex estimated by cryoSPARC 3.1. (d) The models and the corresponding maps for the representative segments of gp24 and gp25.

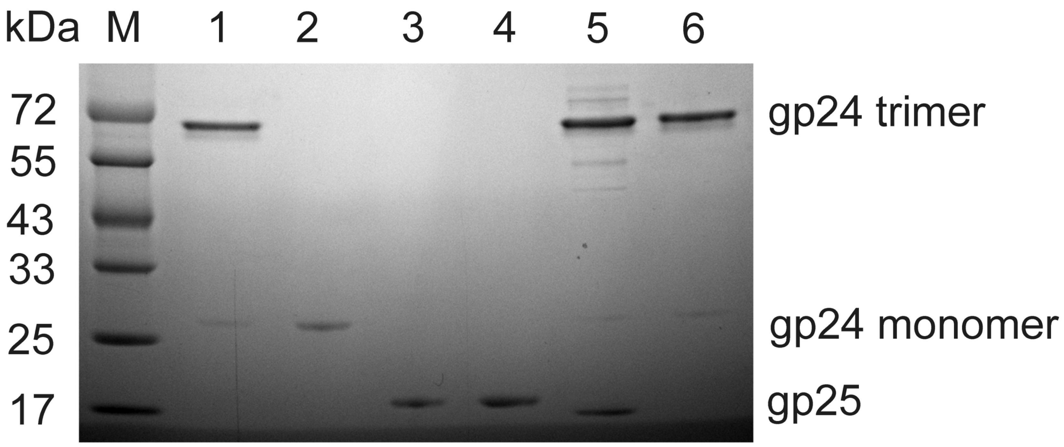

Figure S4. The SDS-PAGE analysis of the purified gp24, gp25 and gp24-gp25 complex. The protein samples were treated with β-Mercaptoethanol without boiling. The lane M represents the standard protein marker,whereas the samples 1~6 are the same as in Figure 1b.

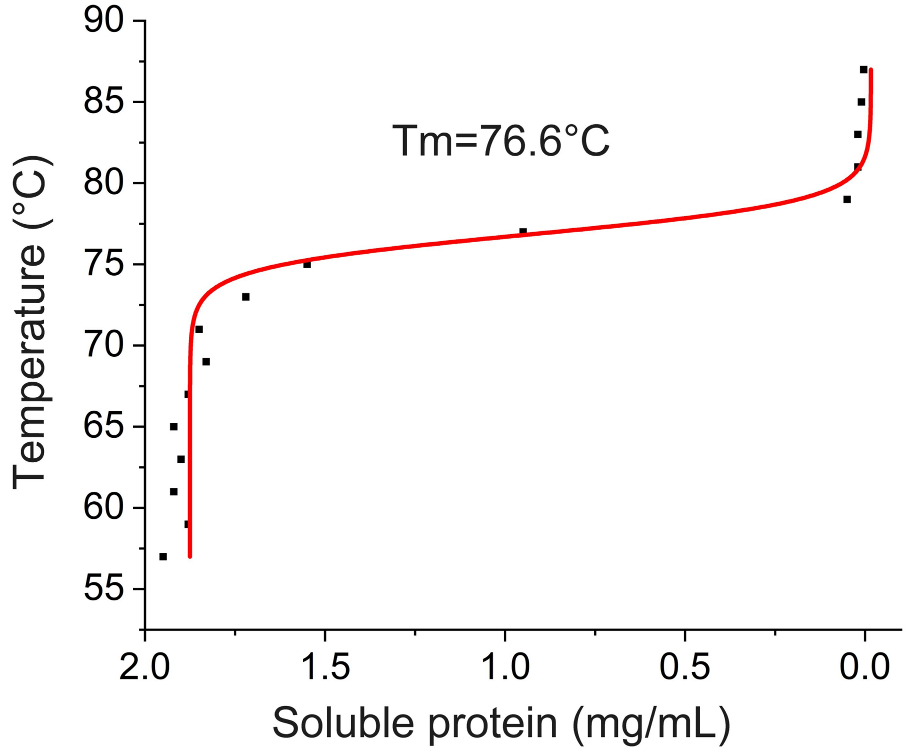

Figure S5. The protein denaturation curve of the gp24 trimer. The calculated Tm value of 76.6°C is labeled.

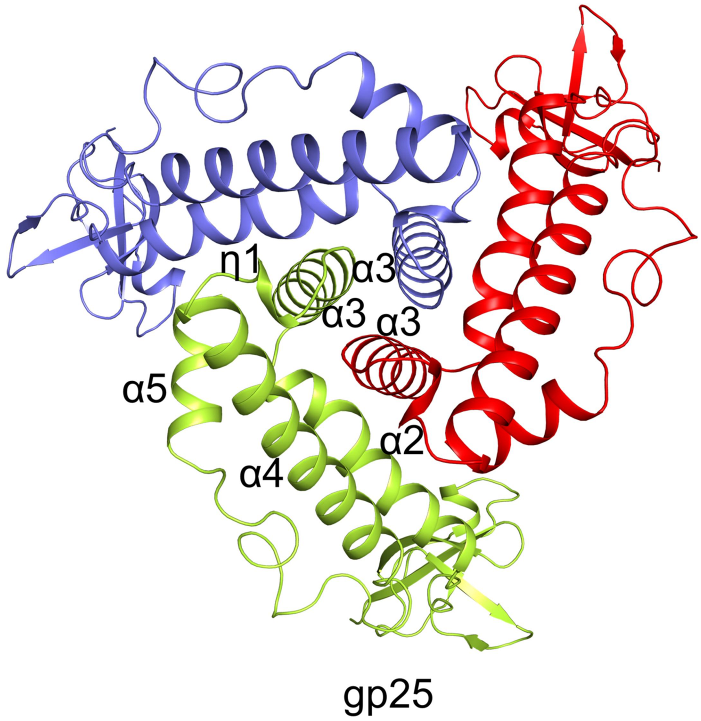

Figure S6. Cartoon representation of gp25 trimer, shown in top view. Three subunits are colored in blue, red and green, respectively.

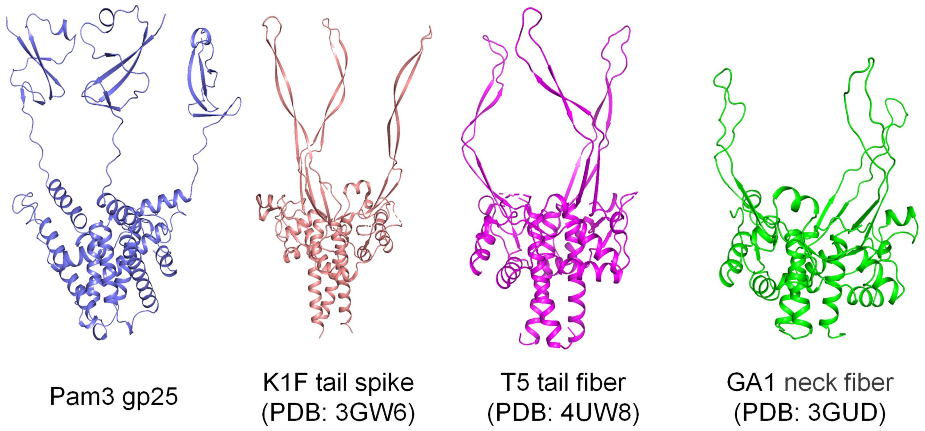

Figure S7. Structures of gp25 and the intermolecular chaperone domains from different phages. The structures are shown in cartoon and colored differently.

Table S1. The mass spectrometry analysis of the intact Pam3 virions.

Gene product	Mw (kDa)	Length (AA)	Coverage	PSMs	Unique Peptides	Predicted protein
**gp6**	17.2	166	98	1017	18	Cement
**gp7**	34.1	310	67	949	33	Major capsid
**gp3**	69.7	621	72	505	37	Portal
**gp16**	62.3	588	66	325	31	Tape measure protein
**gp14**	15.3	142	80	144	8	Tube
**gp13**	41.8	384	65	138	25	Sheath
**gp18**	11.7	106	69	116	7	Plug
**gp22**	32.6	299	63	99	10	Baseplate wedge
**gp23**	26.2	238	50	85	9	Baseplate wedge
**gp4**	29.8	268	55	83	12	Phage minor capsid protein
**gp21**	13.9	125	70	77	7	Sheath initiator
**gp20**	23.5	221	72	70	12	Spike
**gp9**	13.9	131	50	68	6	Adaptor
**gp24**	27.3	280	33	66	5	Tail fiber
**gp10**	16.6	152	45	55	6	Hypothetical protein
**gp17**	19.8	191	57	51	11	Tube initiator
**gp12**	17.7	156	62	49	11	Terminator
**gp5**	377	40.7	32	44	10	Prohead protease
**gp11**	12	110	60	41	6	Connector
**gp19**	25.1	229	62	34	9	Hub
**gp27**	25.6	233	11	2	2	Hypothetical protein
**gp57**	14.8	138	11	1	1	Deoxycytidylate deaminase
